# NUTRITIONAL RISK IS ASSOCIATED WITH LOW BACK PAIN AMONG OLDER ADULTS: RESULTS FROM THE UAB STUDY OF AGING

**DOI:** 10.1093/geroni/igz038.2628

**Published:** 2019-11-08

**Authors:** David R Buys, Marion W Evans, Richard E Kennedy, Julie Locher, Katie Buys, Cynthia J Brown

**Affiliations:** 1 Mississippi State University, Starvkille, Mississippi, United States; 2 University of Alabama at Birmingham, Birmingham, Alabama, United States

## Abstract

Poor nutritional status is associated with adverse health outcomes across the life course, affecting older adults’ ability to maintain overall well-being, limiting physical strength, and affecting mobility. International research has demonstrated associations between nutritional risk and general musculoskeletal pain; however, no research has explored relationships between nutritional risk and low back pain. Using the University of Alabama-Birmingham Study of Aging, we examined this relationship among 1000 community-dwelling older Alabamians (65+years). We used the DETERMINE Checklist, a well-validated nutritional risk assessment and assessed presence and severity of low back pain over the past 4 weeks. We completed univariate and bivariate analysis and multivariate logistic regression, adjusting for factors significant in the bivariate analyses: sex, body mass index, depression, and co-morbidities. More than half of the participants were at nutritional risk (55.2%). In multivariate analyses, one point increases in nutritional risk were associated with a 14% increase in the likelihood of low back pain 95% CI (1.087,1.213); in categorical analyses, moderate nutritional risk and high nutritional risk were associated with an increase in likelihood of low back pain [46% (95% CI 1.07,2.02) and 164% (95% CI 1.80,3.94), respectively]. While this cross-sectional analysis should be interpreted cautiously, it further emphasizes the importance of nutritional health for older adults. Clinicians treating patients with low back pain or nutritional risk may consider assessing for the other condition. When nutritional risk is detected, clinicians should refer to services such as counseling with a registered dietitian or to a social worker for assistance identifying community-based nutritional supports.

